# What Are the Complications, Success and Survival Rates for Autotransplanted Teeth? An Overview of Systematic Reviews and Metanalyses

**DOI:** 10.3390/healthcare10050835

**Published:** 2022-05-01

**Authors:** Ashutosh Kumar Singh, Nikita Khanal, Nisha Acharya, Md Riasat Hasan, Takashi Saito

**Affiliations:** 1 Department of Oral & Maxillofacial Surgery, Tribhuvan University Teaching Hospital, Maharajgunj Medical Campus, Institute of Medicine, Kathmandu 44600, Nepal; dr.ashutosh@iom.edu.np; 2 Dental Surgeon, Ek EK Paila Foundation, Kathmandu 44600, Nepal; drnikitakhanal@gmail.com; 3 Department of Conservative Dentistry and Endodontics, Tribhuvan University Teaching Hospital, Maharajgunj Medical Campus, Institute of Medicine, Kathmandu 44600, Nepal; menishaacharya@gmail.com; 4 Division of Clinical Cariology and Endodontology, Department of Oral Rehabilitation, School of Dentistry, Health Sciences University of Hokkaido, Hokkaido 061-0293, Japan; t-saito@hoku-iryo-u.ac.jp

**Keywords:** autotransplantation, survival rate, success rate, complications, resorption

## Abstract

Background: Autotransplantation is the surgical repositioning of a tooth within the same patient. It can be thought of as the controlled avulsion and re-implantation of a tooth and can be a viable alternative to other dental rehabilitation options. This review aimed to evaluate the survival rate (SR), major complications such as ankylosis rate (AR) and infection-related root resorption (RR), and overall success and failure rate (FR) in autotransplanted teeth. Methods: Six databases were accessed up to January 2021 to obtain all systematic reviews and meta-analyses (SRs and MAs). Study selection: After title and abstract reading, data extraction was performed from eligible SRs. The methodological quality was calculated for the included SRs using the risk of bias in systematic reviews (ROBIS) tool. Results: Six SRs were included in this review. The overall failure rate ranged from as low as 2.0% to 10.32%. The 1-year survival was very high (97.4–98.0%). The 5-year survival rate ranged from 81 to 98.2%. Major complications of AR ranged from 1.2 to 6.2%, and RR ranged from 2.1 to 10.4%. Conclusion: The overall findings from these SR and MA are promising; however, all the SRs include only single-arm prospective or retrospective studies, the SRs are of overall low methodological quality, and for the heterogeneity of the included SRs, well-designed comparative studies with a long-term follow-up are recommended.

## 1. Introduction

Autogenous tooth transplantation (ATT), or autotransplantation, is the surgical movement in one individual of a vital or endodontically treated tooth from its original location in the mouth to another site [1,2]. Tooth loss as a result of dental caries along with trauma is the most common indication, especially when mandibular and maxillary first molars are involved.

Autogenous tooth transplantation was first documented in 1954 by M.L. Hale [3,4]. The major principles of his technique are still followed today. Initial results suggested only a 50% success rate, and there was little widespread acceptance of the technique [5,6]. Recent developments in the understanding of the nature of the periodontal ligament and cementum and the need for careful atraumatic extractions, use of systemic antibiotics, splinting techniques, and endodontic therapy, have led to a considerable improvement in the success rate and an increase in popularity [7,8,9,10,11,12]. The science of autotransplantation has progressed, as evidenced by the high success rates reported in studies over the last decade [13,14,15,16,17,18,19,20,21,22,23]. These studies demonstrate that an autotransplantation is a viable option for tooth replacement for carefully selected patients [24,25,26,27,28].

Even though ATT is now considered a viable solution for tooth replacement, there are many prognostic factors that affect the overall success and survival of these teeth. Stage of root completion, type of donor and recipient tooth, operative technique and handling of the donor’s tooth, recipient site preparation, use of perioperative systemic antibiotics, and adjunctive procedures such as root surface treatment, ex vivo root canal treatment (RCT), type and duration of splinting are some of the factors that have been reported to affect the prognosis [29]. There is a need to analyze the literature and present contemporary and cumulative evidence regarding these prognostic indicators that could potentially alleviate the complications and failure associated with ATT.

Several systematic reviews (SR) and meta-analyses (MA) were conducted, but varied conclusions were often reported [29,30,31,32,33,34]; therefore, a review of SRs and MAs should consolidate the survival rate (SR), major complications such as ankylosis rate (AR) and infection-related root resorption (RR), and overall success and failure rate (FR) in autotransplanted teeth. This should help clinicians decide and counsel the patients regarding the choice of autogenous transplantation. The purpose of this paper was to systematically review all SRs and MAs that have reported the overall FR, SR, AR, and RR, present the overall evidence and point out deficiencies in the existing body of evidence.

## 2. Materials and Methods

An overview of systematic reviews was conducted following the PRISMA statement, and the protocol was registered on PROSPERO (CRD42021256798). The PICOS criteria used for study selection was: 

**Population**: patients who underwent autotransplantation (anterior or posterior, open apex vs. closed apex) of any tooth for any indication (caries vs. trauma).

**Intervention**: autotransplantation of tooth.

**Comparison**: none (Not applicable for outcome and survival analysis review).

**Outcomes**: survival rate (defined clinically as no more than grade 1 mobility and not associated with any pathology); inflammation related external root resorption; ankylosis.

**Setting**: systematic reviews with meta-analyses of primary studies with the selected outcomes of autotransplantation.

### 2.1. Search Strategy

The PubMed, Cochrane Library, Google Scholar, clinicaltrial.gov, and Ovid Embase databases were considered up to May 2020. English language publications with no restriction on publication date were applied. The PubMed search strategy is provided in Table 1. This strategy was modified and used for all other databases. A reference list of included studies was also searched, and experts were counseled regarding any valuable missed studies.

### 2.2. Review Selection

Two investigators (A.K.S. and N.K.) independently conducted the electronic search. Titles and abstracts were screened, assessing the selected reviews in parallel for eligibility. When missing information persisted, full-text reading was established before the final decision. Any discrepancies between the two authors were discussed and judged by a third author (NA). The inclusion criteria concerned systematic reviews and meta-analysis that allowed for the extraction of data on the survival rate (SR), major complications such as the ankylosis rate (AR) and infection-related root resorption (RR), and overall failure rate (FR) in autotransplanted teeth. The exclusion criteria were narrative and scoping reviews as well as systematic reviews without statistical analysis.

### 2.3. Data Extraction

The same two investigators (AKS and NK) independently conducted the data extraction from the eligible reviews. The recorded information was the following: author, publication date, study design (SR or MA), number of included studies, country of the included studies, number and type of included donor and recipient’s tooth, methodological data, quality assessment of the primary studies, outcomes, review results, and author’s conclusion. Any discrepancy was solved by consultation with a third investigator (NA).

### 2.4. Analysis of Methodological Quality

Two investigators (AKS and NA) analyzed the methodological quality of each SR, calculating the score using ROBIS tool. The ROBIS tool was used to provide a tabular graphical display of results [35].

## 3. Results

### 3.1. Search Results and Review Selection

**Figure 1:**
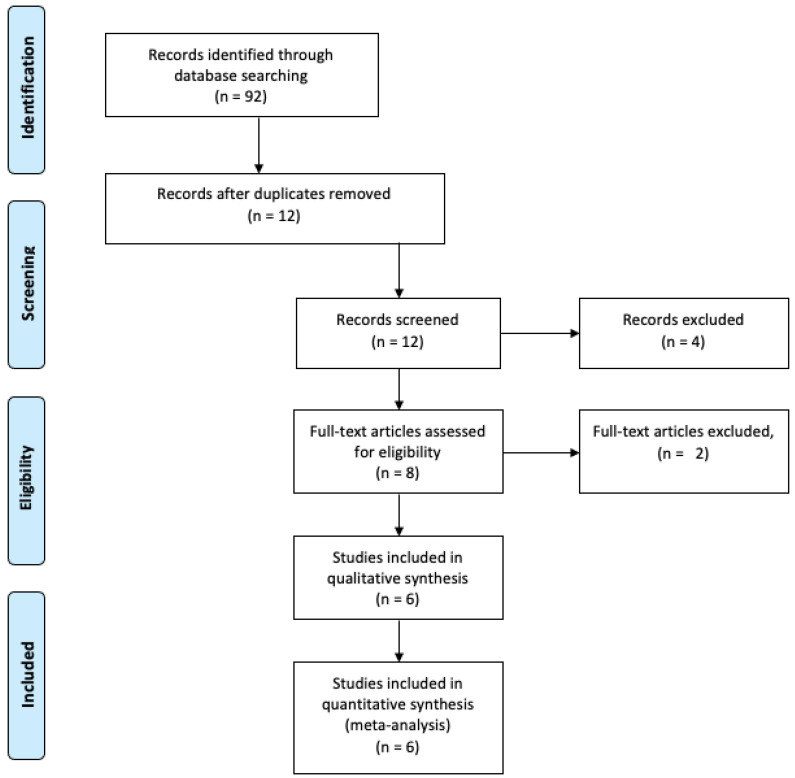
Study selection flow diagram.

The electronic search was performed until January 2022, producing a total of 92 records from six different databases: PubMed, *n* = 28; Cochrane Library, *n* = 12; Google Scholar, *n* = 16, Embase, *n* = 36, Clinicaltrial.gov, *n* = 0. Titles and abstracts were screened after the removal of duplicates, and a total of 12 potentially significant records were assessed. Eight full-text articles were identified for eligibility. After full-text reading, two studies were excluded because they did not meet the inclusion criteria. A list of all the excluded studies is provided in the flow diagram showing the SR selection (Figure 1), and, finally, six SRs were included in this review. All six SRs also conducted MAs.

### 3.2. Study Characteristics

Among the six included systematic reviews and meta-analyses, three were from Europe [29,32,34], two from South America [30,31], and one from Asia [33]. All the reviews were published in English. The primary study search was without language restriction for two reviews [29,30], including English, Spanish and Portuguese for one review [31] and English language only for three reviews [32,33,34]. One review included primary studies on only the ATT in the anterior maxilla, [34] two reviews included only ATT with open apex (incomplete root formation) [31,32], one review included teeth with closed apex only (complete root formation) [33], and two reviews [29,30] included any donor tooth/any recipient site, one of which [30] presented long-term prognosis of the ATT. Study characteristics are provided in detail in Table 2. All the reviews included only single-arm prospective or retrospective cohort studies or case series with at least 10 ATT since comparative quasi-experimental or proper randomized controlled or uncontrolled clinical trials have not been performed to date. Five reviews [29,30,32,34] declared no conflict of interest whereas one review [31] did not declare. Funding was not available for one review [32], institutional and self-supported for two reviews [30,33], research grant for one review [31], and not declared by two reviews [29,34].

GRADE approach for evidence certainty and clinical recommendation was performed by only one study [29]. Risk of bias analysis reported low-quality evidence and weak methodology in primary studies in all the reviews. The primary reason cited for weak methodology was the small sample size, lack of control, and the possibility of selective reporting in most of the primary studies with retrospective design.

### 3.3. Analysis of Methodological Quality

Only one study had a high level of quality [30]. Five included SRs had a low level of quality [29,31,32,33,34]. The specific evaluation of the checklist recorded the following as the most critical issues: the absence of details for excluded studies, protocol not registered, weak calculation, and failure to discuss the effect the risk of bias of the included studies had on the overall result. The ROBIS risk of bias is displayed visually in Table 3 below.

### 3.4. Success and Failure Rate

In the MA by Chung et al. [33], the summary estimate of the annual FR was 2.0% [95% confidence interval (CI): 1.2–3.2%], based on 25 studies. The reported percentages of survival from individual studies fell within a wide range of 30–100%. Some studies also presented the 1-year SR. All reported 1-year SRs were >88%. Based on the meta-analysis, they found that the estimated FR of autotransplanted teeth with complete root formation was only 2.0%; the estimated 1-year and 5-year SRs were 98.0% and 90.5%, respectively. In the MA by Almpani et al. [29], the pooled FR in the included studies was found to be 7.8% (95% CI: 4.7–10.9%), based on 15 studies. In the MA by Machado et al. [30], the survival rate was mentioned in four studies and ranged from 75.3% to 91%. The meta-analysis showed a significant effect size of 81% (95% CI: 73.8–86.6%) (*p* < 0.0001). Heterogeneity among the studies was low. In the MA by Atala-Acevedo et al. [31], seventeen studies were included as evidence to determine the success rate of autotransplantation with an open apex. The success rate of the studies was 89.68% (95% CI 86.77 to 92.59%). The success rate was high, although the heterogeneity of 64.6% was substantial. The autotransplantation survival rate in the 15 studies included was 98.21% (95% CI, 96.99 to 99.44), with a low heterogeneity of 25.3%. In the MA by Akhlef et al. [34], survival rates ranged between 93% and 100% (weighted mean: 96.7%, median: 100%) after from 9 months to 22 years of observation (median: 8.75 years), based on 11 studies.

In the MA by Rohof et al. [32], the weighted estimated yearly success rate was 96.6% (95% CI, 94.8–97.8). No heterogeneity was found (Q = 8.24; *p* = 0.99; I2 = 0.0%), based on 23 studies. The survival rate after 1 year was reported in 26 articles, with the average weighted survival rate of 97.4% (95% CI, 96.2–98.2%). No heterogeneity was found across these studies (Q = 13.66; *p* = 0.98; I2 = 0.0%). The survival rate after 5 years was reported in 11 articles, with the average weighted survival rate of 97.8% (95% CI, 95.0–99.0%). The data on 5-year survival showed 19.6% heterogeneity (Q = 12.4; *p* = 0.26), which can be considered low. The survival rate after 10 years was reported in six articles, with the average weighted survival rate of 96.3% (95% CI, 89.8–98.7%). The heterogeneity was 56.8%, which can be considered substantial (Q = 11.6; *p* = 0.04). The weighted estimated survival rate per year was 98.2% (95% CI, 96.4–99.1%). No heterogeneity was found (Q = 6.2; *p* = 0.99; I2 = 0.0%).

### 3.5. Ankylosis Rate

In the MA by Chung et al. [33], the results showed that the estimated first-year AR was 1.2% (95% CI: 0.5–3.2%). However, two aberrant studies demonstrated 100% ARs. Seven studies did not report the occurrence of ankylosis. In the MA by Almpani et al. [29], the pooled AR in the included studies was found to be 76.2% (95% CI: 4.5–7.8%), based on 11 studies. In the MA by Machado et al. [30], four studies reported the percentage of replacement resorption (ankylosis) in transplanted teeth, ranging from 4.2% to 18.2%. The meta-analysis showed high heterogeneity among studies, and after the sensitivity analysis, the heterogeneity decreased considerably, and a significant effect size of 4.8% was observed (*p* < 0.0001). In the MA by Rohof et al. [32], the weighted estimated ankylosis per year was 2.0% (95% CI, 1.1–3.7%).

### 3.6. Infection-Related Root Resorption Rate

In the MA by Chung et al. [33], the estimated first-year RR from 25 included studies was only 2.1%. Two of the included studies presented an RR of >50%. On the other hand, they considered four studies that did not report the occurrence of infection-related root resorption as no occurrence. In the MA by Almpani et al. [29], the pooled RR in the included studies was found to be 10.4% (95% CI: 7.0–13.7%), based on 19 studies. In the MA by Machado et al. [30], when surface resorption, inflammatory resorption, and external root resorption were considered together in the meta-analysis, a significant effect size of 19% was observed (*p* < 0.0001). The heterogeneity among studies, nevertheless, was extremely high, and after the sensitivity analysis, the heterogeneity decreased considerably, and a significant effect size of 4% was observed (*p* < 0.0001). In the MA by Rohof et al. [32], the weighted root resorption per year was 2.9% (95% CI, 1.5–5.5%).

### 3.7. Other Reported Outcomes

In the MA by Almpani et al. [29], hypermobility was 8% (95% CI: 4.1, 11.9) based on eight studies, pulp necrosis was 34.3% (95% CI: 21.1, 47.4) based on 10 studies, and pulp obliteration was 53.4% (95% CI: 28.3, 78.5) based on five studies. In the MA by Machado et al. [30], all studies reported the pulp condition of the transplanted teeth. In the MA by Rohof et al. [32], the weighted pulp necrosis per year was 3.3% (95% CI, 1.9–5.6%). The study outcomes and results are provided in detail in Table 3.

### 3.8. Subgroup Analysis Based on Factors Could Potentially Mediate the Prognosis of ATT

Chung et al. [33] reported that the estimated FR was higher in the absence of SA, suture splinting, wire splinting ≤ 14 days, and posterior donors. The estimated RR was higher in the absence of SA, endodontic treatment within post-operative 14 days, and anterior/premolar donors [33]. The estimated AR was higher with wire splinting and premolar donors. The stage of development of the root and the autotransplantation receptor site showed no statistically significant differences [33]. CM Rohof et al. reported a higher success and survival rate with the maxillary recipient site compared to the mandible site and premolar recipient site compared to the molar recipient site. They also reported higher success and survival rates with premolar donors than with molar donors. Ankylosis rate and root resorptions were higher in molars compared to premolar donors, but the pulp necrosis rate was higher with the premolars [32]. Almpani et al. reported lower failure rates with open apex compared to closed apex and suture splinting compared to wire splinting. The calculated NNT is seven; for every seven ATT with a suture splint, one failed transplant could be prevented compared to transplants with a wire-composite splint. For root development, the calculated NNT is six; for every seven ATT with open apex, one failed transplant could be prevented compared to transplants with closed apex [29]. Atala et al. reported a higher survival rate of teeth at stages three and four of root completion compared to stages one, two, and five (closed apex), premolars compared to molars, and in the maxilla compared to the mandible. Ankylosis was also found to be an indicator of the failure of the transplant [31]. The subgroup analysis is tabulated in Table 4.

### 3.9. Publication Bias

Analysis of publication bias was performed in only two of the SR and MAs. Machado et al., in their Funnel plot analysis, showed a tendency toward the publication of studies with high survival rates [30]. Almpani et al. reported Funnel plot asymmetry for all outcomes; the Eggers test was significant for FR, RR, and pulp necrosis outcomes [29].

### 3.10. Analysis of Primary Studies Overlap in the Reviews

The overlap of primary studies was analyzed using a citation matrix where the rows represent the primary studies and columns represent the reviews [36,37]. A total of 91 primary studies were included in the reviews. Three studies were overlapping in four reviews, eight studies were overlapping in three reviews, 22 studies were overlapping in two reviews, and 58 studies did not overlap. The citation matrix is presented in Table 5. The corrected coverage area was calculated to quantify the overlap using the formula: CCA = N-r/rc-r, where N = total number of included studies, including double counting (total check marks), r = total unique primary studies (number of rows), and c = total reviews (number of columns). The calculated CCA was 0.08, which means insignificant overlap (CCA 1–5 is slight, 6–10 is moderate, 11–15 is high, and >15 is very high overlap). Not surprisingly, fifteen primary studies are overlapping in the review by Atala et al. and Evelyn Rohof et al., as both these reviews include ATT with open apex only. The calculated CCA for these two similar reviews is also slight (0.33). Hence, the overestimation of the effect is potentially low when combining these reviews due to only a slight primary study overlap.

## 4. Discussion

This review aimed to answer the question: “What is the survival and success/failure rate of autotransplanted tooth, and what is the rate of major complications like AR and RR?” Six SRs and MAs were included in this study, but their methodological heterogeneity and the slight overlap of studies precluded an umbrella meta-analysis. In order to assess the quality of each SR, the AMSTAR-2 tool and ROBIS tool were used. The qualitative analysis of the included SRs allowed for the SR, FR, AR, and RR to be summarized. The overall failure rate ranged from as low as 2.0% to 10.32%. The 1-year survival was very high (97.4–98.0%). The 5-year survival rate ranged from 81 to 98.2%. Major complications of AR ranged from 1.2 to 6.2%, and RR ranged from 2.1 to 10.4%. Considerable heterogeneity exists across SRs, with differences in terms of donor tooth, recipient site, and the stage of root formation. In this current review, high methodological quality was assessed for only one SR [30], whereas five others were of low quality [29,31,32,33,34]. The evidence from included reviews was undermined by the absence of studies directly comparing various morphology, areas, modality, and perioperative techniques for ATT, as only single-arm studies were available for analysis. All the reviews have conceded this limitation in their discussion and have provided the explanation that it will be unethical to perform an RCT knowingly when one treatment is supposed to be superior to the other; thus, comparative controlled trials were not feasible. Thus, we should be cautious, as suggested by the review authors, that the findings should be interpreted in the light of the limitations under which the data were aggregated and compared, as only an indirect comparison was possible from multiple single-arm cohorts, which were then post hoc considered as reference and comparator group for statistical analysis. For this same reason, we did not perform an umbrella meta-analysis as this would further increase the bias of performing indirect comparison among groups that come from disparate studies and are not matched for confounders.

Based on the subgroup analysis of different donor tooth and recipient sites, evidence suggests that anterior donor and recipient sites have a higher survival rate compared to molars. This has been attributed to the ease with which the single-rooted anterior donors can be atraumatically harvested, causing less damage to the PDL, which is one of the most important prognostic factors for the success of the transplant. Maxillary transplants were found to have higher survival than mandibular ones, probably because of highly porous alveolar bone and abundant blood supply, as well as less direct occlusal loading of maxillary teeth compared to mandibular teeth. The results of subgroup analysis based on various techniques utilized along with ATT hint towards the use of suture splinting rather than wire splinting, performing ATT using donor tooth with open apex, performing ex vivo RCT, or RCT within 14 days of transplant, and using perioperative systemic antibiotics. Teeth with open-apex have been reported to allow neurovascular ingrowth after transplantation which might explain their post-transplant vitality and better survival rate. Wire-splinting is more rigid than suture splinting, thus allowing lesser micro-motion of the ATT in the post-operative procedure. Though it may provide more stability, the lack of physiological stimulus may prevent the optimum level of tissue signaling from initiating adaptation of the ATT in the recipient site and may lead to avascular necrosis due to rigid pressure applied by the wire splint. Another interesting finding is higher survival of teeth where RCT was initiated within 14 days, and systemic antibiotics were used. Antibiotics definitely would have reduced the incidence of infection post-ATT, which may lead to periodontal tissue destruction and loosening of transplant. The prompt initiation of RCT may help to prevent pulpal infection and necrosis, which, if uncontrolled, is one of the main reasons for root resorption and ATT failure.

Autotransplantation, or the process of moving a tooth from one site to another in the same person, is not a new technique in the history of dentistry but has varying success and failure rates in the dental literature [3,37,38,39]. This surgical method can be used to replace missing, avulsed, traumatized, or grossly decayed, unrestorable teeth with high survival rates (91.3%), particularly in adults or children [40,41,42]. Ironically this treatment option is grossly overlooked, and the use of dental implants or fixed prostheses is widely used instead. Dental implants are not a good option for patients until the completion of growth, which is 17–19 years for girls and 19–21 years for boys [43]. However, loss of a tooth is more prevalent in young adults due to trauma or caries. Autotransplantation could hence be a very good alternative for these patients, with lots of advantages, such as preservation and normal functioning of periodontal ligament tissues and alveolar bone volume, along with proprioception, better esthetics, and higher resistance to occlusal load [16]. Moreover, even in the case of external root resorption or replacement resorption, the bone and soft tissue volume will be preserved, favoring subsequent implant placement [16,44]. Now that it is established that the success and survival rate of ATT is overalls high, and AR and RR are considerably low, direct comparative prospective studies, preferably randomized and blinded, should be performed to evaluate the factors that can further decrease complications and failure rate, and increase overall survival over a long period of time.

Recently, various methods have been incorporated into ATT to increase its success and survival rates. One such method is the use of 3D-printed guiding templates/replicas and GBR, which significantly reduces the extra-oral time of the tooth, resulting in less drying and trauma for the PDL cells [45,46,47,48,49,50,51,52,53]. Additionally, ATT can be performed by creating artificial tooth sockets with the use of cone-beam computed tomography (CBCT) and 3D-printed donor tooth replicas. The incorporation of this modern technology in the ATT makes this procedure less technique-sensitive, reduces extra-oral time and iatrogenic damage, predicts a good prognosis, and facilitates a successful outcome [45,49,54,55,56]. However, occlusion reduction, splinting, and root canal treatment are still indicated to reduce RR.

It has also been observed that, with proper case selection and appropriate treatment, ATT can be successfully performed with fully formed third molars as donors in both immediate extractions as well as surgically created sockets [57]. Previously, external inflammatory or internal root resorption was considered to be a major reason for the failure of the ATT tooth [58]. However, this kind of resorption can easily be controlled with the timely initiation of endodontic therapy, or even after the initiation of resorption, endodontic treatment and long-term calcium hydroxide dressings were found to treat these conditions successfully, without any signs of mobility, pocket, radiolucency, tenderness to percussion, or any other pathology [59]. Similarly, a minimal amount of trauma and extra-oral time are crucial for preserving intact and viable periodontal cells, which aid in the healing of the transplanted teeth without root resorption, especially the replacement resorption/ankylosis [60]. Thus, the horizon of the scope of autotransplantation is becoming broader, with many possibilities, newer advancements, and a very predictable and favorable outcome. The literature search suggests that autotransplantation is a very cost-effective, viable, immediate treatment option and a biological solution that can be provided to adolescent patients with a missing tooth when a suitable donor tooth is present, with a high success rate [28].

### 4.1. Limitations and Strengths of This Overview

This overview of reviews has some limitations. We included all the autotransplanted teeth for any indication to accrue a cumulative complication and survival rate, but that also means that we have evidence from essentially heterogeneous sources. There is no considerable difference in the technique of autotransplantation, but the clinical protocol, systemic antibiotics, and local procedures to increase viability differed among the primary studies included in the reviews. The included reviews were found to have low methodological quality due to the exclusion of important steps from PRISMA guidelines, and the meta-analyses were all based on indirect group comparisons from disparate studies that were not matched for confounders. Our literature search included results from only six databases and the English language only and did not include gray literature, pre-prints, or commercial reports, which might have added to the evidence if available. The primary study overlap was low between the reviews; thus, we can say that at least the cumulative results are not overestimated, although indirectness of comparison brings down the level of confidence in the results.

### 4.2. Implications for Practice and Research

Based on the comprehensive and cumulative evidence from available systematic reviews and meta-analyses, the success rate (SR) of the autotransplanted tooth shows promising results, above 90%, even with long-term follow-up and a survival rate up to 98%. In the era of implant-driven dentistry, preserving natural teeth is more economical, biological, and esthetic. Natural teeth with viable periodontium are more physiological than a dental implant, which is similar to ankylosed teeth. However, the success of autotransplantation depends upon various prognostic factors, such as the age of the patient, stage of root development at the time of transplantation, splinting method and duration of transplanted teeth, amount of trauma perceived by the PDL at the time of extraction, administration of systemic antibiotics, initiation of endodontic treatment, compliance of the patient, etc. Thus, these factors determine the success and failure, with either root resorption or replacement resorption, of the transplanted tooth. Hence, more studies are recommended on various prognostic indicators to obtain accurate protocols for autotransplantation.

## 5. Conclusions

The overall findings from these SR and MAs are promising. The autotransplanted tooth has a low failure rate, high survival rate, and low complication rate, but the overall evidence is of low to moderate certainty, and all the SRs are based on only single-arm prospective or retrospective studies. Further research on the evaluation of prognostic indicators and factors that affect the success of ATT is recommended.

## Figures and Tables

**Table 1:** Search Strategy for PubMed which was modified and used for other databases.

Query	Filters	Search Details	Results
(teeth OR tooth) AND (((autotransplant) OR (autotransplant *)) OR (autotransplantation))	Systematic Review	((“teeth s”[All Fields] OR “teeths”[All Fields] OR “tooth”[MeSH Terms] OR “tooth”[All Fields] OR “teeth”[All Fields] OR “tooth s”[All Fields] OR “tooths”[All Fields] OR (“teeth s”[All Fields] OR “teeths”[All Fields] OR “tooth”[MeSH Terms] OR “tooth”[All Fields] OR “teeth”[All Fields] OR “tooth s”[All Fields] OR “tooths”[All Fields])) AND (“autografts”[MeSH Terms] OR “autografts”[All Fields] OR “autotransplant”[All Fields] OR “autotransplants”[All Fields] OR “autotransplanted”[All Fields] OR “autotransplanting”[All Fields] OR “autotransplant *”[All Fields] OR (“autotransplantion”[All Fields] OR “transplantation, autologous”[MeSH Terms] OR (“transplantation”[All Fields] AND “autologous”[All Fields]) OR “autologous transplantation”[All Fields] OR “autotransplantation”[All Fields] OR “autotransplantations”[All Fields]))) AND (systematicreview[Filter])	28

**Table 2:** Included reviews arranged according to timeline of publication with their characteristics.

Author and Year Published	Country of Study	Study Design	Database Search	Language and Time Period Restriction	Included Study Character
Chung et al., 2014 [33]	Taiwan	SR and MA	PubMed, Google Scholar, Scopus	Language: English Date; between 1771 and 28 February 2013	a sample size of at least 10 permanent transplanted teeth. complete root formation and a closed apical foramen. at least a 1-year follow-up period
Almpani et al., 2015 [29]	Germany	SR and MA	MEDLINE, LILACS, Scopus, Ovid database, BioMed Central, ProQuest, Cochrane Library, African Journals Online, Lippincott Williams and Wilkins (LWW), Bibliografia Brasileira de Odontologia, Google Scholar Beta, Wiley Online Library, Elsevier Book Series and Health Sciences	Language: No restriction Date: up to November 2012	studies providing information regarding the success/survival rate of autologous transplantation of teeth in the short- or/and long-term
Machado et al., 2015 [30]	Brazil	SR and MA	PubMed, Scopus, Web of Science, Lilacs, and The Cochrane Library	Language: No restriction Date: (1 January 1990 to 7 July 2014).	studies reporting at least one of the following: survival rate, pulp condition, mobility, presence of ankylosis, and root resorption of autotransplanted teeth with complete or incomplete root formation. Follwup >6 years.
Akhlef et al., 2016 [34]	Denmark	SR and MA	PubMed	Language: English Date: NR	Autotransplantation to the anterior maxilla. Studies including ≥10 teeth.
Atala et al., 2017 [31]	Chile, Spain	SR and MA	MEDLINE, EMBASE, LILACS, SciELO	Language-English, Spanish, Portuguese Date: January 1997 to August 2015	open apex with or without preparation of the socket. a minimum follow-up period of 12 months.
C.M. Rohof et al., 2018 [32]	Netherland	SR and MA	PubMed, EMBASE, Web of Science, Cochrane Library	Language-English Date: all data published until July 2016	involving 5 or more participants and at least 10 permanent transplanted teeth. incomplete root formation Open apex reported or deducible success rates at least 1-year mean follow-up period

**Table 3:** ROBIS Results for risk of bias analysis in the included systematic reviews.

Review	Phase 2				Phase 3
	1. Study Eligibility Criteria	2. Identification and Selection of Studies	3. Data Collection and Study Appraisal	4. Synthesis and Findings	Risk of Bias in the Review
Machado et al. [30]	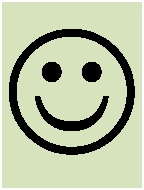	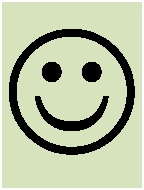	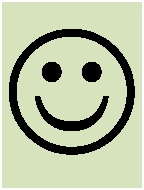	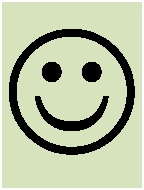	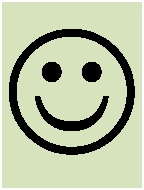
Evelyn Rohof et al. [32]	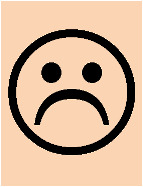	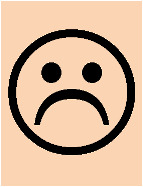	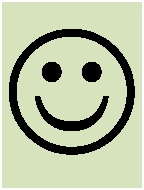	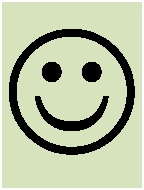	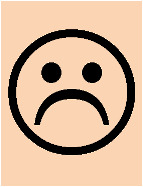
Chung et al. [33]	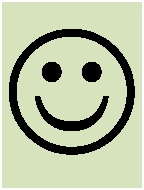	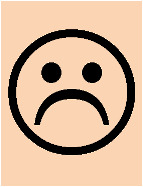	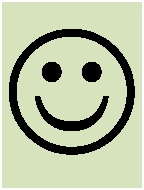	?	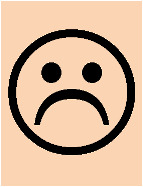
Atala-Avecado et al. [31]	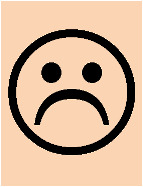	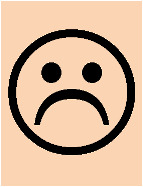	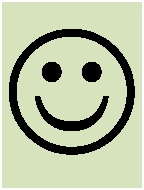	?	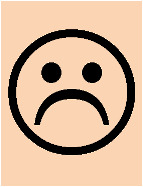
Almpani et al. [29]	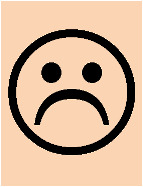	?	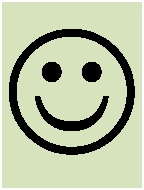	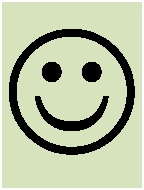	?
Akhlef et al. [34]	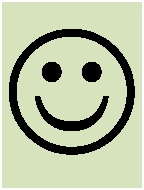	?	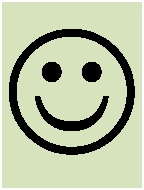	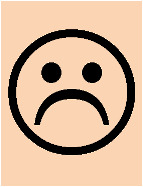	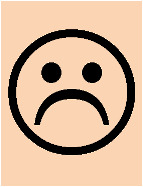

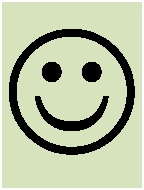
 = low risk; 
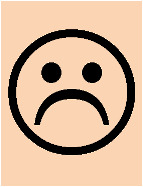
 = high risk; ? = unclear risk.

**Table 4:** Details of subgroup analysis and their results based on prognostic factors in the included reviews.

Author and Year Published Number of Included Studies	Subgroup Analysis	Results and Conclusion
Chung et al., 2014 [33] 26 studies	systemic antibiotics (SAs), endodontic and splinting modalities and donor tooth morphology	Systemic Antibiotics: FR (IRR = 2.5, 95% CI: 0.9–7.2) and Root resorption (IRR = 1.4, 95% CI: 0.2–8.9) Endodontic treatment: FR (IRR = 1.0, 95% CI: 0.2–5.2) and Root resorption (IRR = 2.0, 95% CI: 0.2–9.3) Splinting: FR (IRR = 0.8, 95% CI: 0.1–5.5) Splinting ≥14 days vs. Splinting <14 days FR (IRR = 0.4, 95% CI: 0.1–2.0) Wire splinting vs. Suture splinting AR (IRR = 3.0, 95% CI: 0.0–607.9) Donor tooth: Annual Failure rate anterior (0.6% (95% CI: 0.2–2.3%), premolar 1.6% (95% CI 0.3–9.1%), and molar donors was 3.3% (95% CI: 2.4–4.7%) 1-Year Survival rate anterior, 99.4% (95% CI: 97.7–99.8%), premolar 98.4% (95% CI: 90.9–99.7%) and molar donors was 96.7% (95% CI: 95.3–97.6%) 5-Year Survival rate anterior, 96.9% (95% CI: 89.1–99.2%), premolar 92.3% (95% CI: 62.1–98.6%) and molar donors was 84.3% (95% CI: 78.7–88.6%)
Almpani et al., 2015 [29] 38 studies	Open apex vs. Closed apex Splinting Donor tooth	Open apex vs. closed apex: FR (RR: 0.3; 95% CI: 0.2–0.6) Wire splint vs. suture splint FR (RR: 3.7; 95% CI: 1.1–12.6) NSD for donor tooth, patient age (>20 vs. <20), gender, recipient site and surgical technique.
Atala-Acevedo et al., 2016 [31] 17 studies	Donor tooth, Recipient site, stage of root formation.	Donor tooth (Premolar vs. Molar) FR (OR, 0.46; 95% CI, 0.25 to 0.84) Recipient (Maxilla vs. Mandible) FR (OR, 0.38; 95% CI, 0.09 to 1.60)
C.M. Rohof et al., 2018 [32] 32 studies	donor tooth type recipient site, root development, splinting procedure, splinting duration, orthodontic procedure, and antibiotic regimen.	Survival rate: Premolar as recipient site (98.6%), Molar (97.3%) Premolar donor (98.4%), Molar (97.2%) Success rate: As recipient site; Incisor (98.5%), Canine (97.7%), Premolar (97.8%), Molar (95.1%) Maxillary recipient site (98.5%) vs. Mandibular recipient site (97.3%) Ankylosis rate: Premolar donor (1.9%), Molar donor(2.2%) Root Resorption Premolar donor (1.5%), Molar donor (5%) Pulp Necrosis: Premolar donor (4.4%), Molar donor (2.5%)

Footnotes: NSD = No significant difference, FR = Failure rate; RR = Risk ratio, IRR = Incident risk ratio; AR = ankylosis rate.

**Table 5:** Citation matrix and overlap of primary studies in the included reviews.

Primary Studies	Akhlef 2017	Almpani 2015	Atala 2016	Chung 2014	Evelyn Rohof 2018	Machado 2016	Size of Overlap
1. Andreasen et al., 1990	X						1
2. Bowden et al., 1990	X						1
3. Czochrowska et al., 2000	X		X		X	X	4
4. Gilijamse et al., 2016	X						1
5. Kristerson and Lagerström, 1991	X		X		X		3
6. Kugelberg et al., 1994	X			X	X		3
7. Kvint et al., 2010	X						1
8. Mendoza-Mendoza et al., 2012	X		X		X	X	4
9. Slagsvold et al., 1978	X				X		2
10. Stange et al., 2016	X						1
11. Tanaka et al., 2008	X		X		X		3
12. Ahlberg et al., 1983		X		X			2
13. Akiyama et al., 1998		X					1
14. Akkocaoglu and Kasaboglu		X					1
15. Altonen et al., 1978		X					1
16. Andreasen et al., 1990		X	X		X		3
17. Andreasen et al., 1990		X	X				2
18. Andreasen et al., 1990		X	X				2
19. Andreasen et al., 1990		X	X		X		3
20. Arikan et al. 32 2008		X		X			2
21. Azaz et al., 1978		X		X			2
22. Bauss et al., 2002		X			X		2
23. Bauss et al., 2008		X					1
24. Bauss et al., 2004		X					1
25. Bauss et al., 2004		X					1
26. Bauss et al., 2005		X					1
27. Bauss et al., 2008		X					1
28. Bauss et al., 2008		X					1
29. Bauss and Kiliaridis 2009		X					1
30. Eliasson et al., 1988		X		X			2
31. Kahnberg 1987		X					1
32. Kristerson 1985		X		X	X		3
33. Kristerson et al., 1991		X		X			2
34. Lagerstron and Kristerson 1986		X					1
35. Lundberg and Isaksson 1996		X	X		X		3
36. Marques- Ferreira et al., 2011		X					1
37. Mejare et al., 2004		X		X			2
38. Myrlund et al., 2004		X			X		2
39. Nethander et al., 1988		X					1
40. Nethander 1994		X					1
41. Nethander 1998		X		X			2
42. Ploder et al., 2001		X					1
43. Pogrel et al., 1987		X					1
44. Reich 2008		X					1
45. Sagne et al., 1986		X					1
46. Sobhi et al., 2003		X					1
47. Sugai et al., 2010		X		X			2
48. Thomson et al., 1984		X		X			2
49. Yan et al., 2010		X	X	X	X		4
50. Bauss et al., 2002			X				1
51. Bauss et al., 2004			X				1
52. Bauss et al., 2003			X				1
53. Czochrowska et al.,2000			X		X		2
54. Denys et al., 2013			X				1
55. Dıaz et al., 2014 (17)			X				1
56. Isa-Kara et al., 2011			X	X	X		3
57. Josefsson et al., 1999			X		X		2
58. Kallu et al., 2005			X				1
59. Mertens et al., 2014			X				1
60. Nagori et al., 2014			X		X		2
61. Naranjo et al., 2002			X				1
62. Plakwicz et al., 2013			X		X		2
63. Schutz et al., 2013			X		X		2
64. Vilhjalmsson et al., 2011			X		X		2
65. Forssell and Oksala (1986)				X			1
66. Gault and Warocquier-Clerout (2002)				X			1
67. Hovinga (1969)				X			1
68. Masif and Youseff (1977)				X			1
69. Moss (1968)				X			1
70. Niimi et al. (2011)				X			1
71. Patel et al. (2011)				X			1
72. Reade et al. (1973)				X			1
73. Schatz and Joho (1993)				X			1
74. Sange and Thilander (1990)				X			1
75. Schwartz et al. (1985)				X			1
76. Wang et al. (2007)				X			1
77. Watanabe et al. (2010)				X		X	2
78. Borring-Møller et al.1979					X		1
79. de Carvalho et al. (2014)					X		1
80. Díaz et al., 2008					X		1
81. Gonnissen et al., 2010					X	X	2
82. Hernandez and Cuestascarner 1988					X		1
83. Jonsson and Sigurdsson 2004					X		1
84. Marcusson and Lilja-Karlander 1996					X		1
85. Mertens et al., 2016					X		1
86. Mensink and van Merkesteyn 2010					X		1
87. Nagori et al. (2014)					X		1
88. Schatz and Joho 1992					X		1
89. Shahbazian et al., 2013					X		1
90. Paulsen and Andreasen (1998)						X	1
91. Paulsen et al. (1995)						X	1

X = included in the review.

## Data Availability

Medline (PubMed) and Scopus databases.
